# Improving Human Induced Pluripotent Stem Cell-Derived Megakaryocyte Differentiation and Platelet Production

**DOI:** 10.3390/ijms22158224

**Published:** 2021-07-30

**Authors:** Linda Krisch, Gabriele Brachtl, Sarah Hochmann, André Cronemberger Andrade, Michaela Oeller, Patricia Ebner-Peking, Katharina Schallmoser, Dirk Strunk

**Affiliations:** 1 Cell Therapy Institute, Spinal Cord Injury and Tissue Regeneration Center Salzburg (SCI-TReCS), Paracelsus Medical University (PMU), 5020 Salzburg, Austria; linda.krisch@pmu.ac.at (L.K.); gabriele.brachtl@pmu.ac.at (G.B.); sarah.hochmann@pmu.ac.at (S.H.); andre.cronemberger@pmu.ac.at (A.C.A.); patricia.ebner@pmu.ac.at (P.E.-P.); 2 Department of Transfusion Medicine and SCI-TReCS, Paracelsus Medical University (PMU), 5020 Salzburg, Austria; m.oeller@salk.at (M.O.); k.schallmoser@salk.at (K.S.)

**Keywords:** regenerative medicine, human induced pluripotent stem cells (hiPSC), mesoderm induction, platelets, megakaryocytes, angiogenesis, human platelet lysate (HPL), advanced therapy medicinal product (ATMP), ROTEM

## Abstract

Several protocols exist for generating megakaryocytes (MKs) and platelets from human induced pluripotent stem cells (hiPSCs) with limited efficiency. We observed previously that mesoderm induction improved endothelial and stromal differentiation. We, therefore, hypothesized that a protocol modification prior to hemogenic endothelial cell (HEC) differentiation will improve MK progenitor (MKP) production and increase platelet output. We further asked if basic media composition affects MK maturation. In an iterative process, we first compared two HEC induction protocols. We found significantly more HECs using the modified protocol including activin A and CHIR99021, resulting in significantly increased MKs. MKs released comparable platelet amounts irrespective of media conditions. In a final validation phase, we obtained five-fold more platelets per hiPSC with the modified protocol (235 ± 84) compared to standard conditions (51 ± 15; *p* < 0.0001). The regenerative potency of hiPSC-derived platelets was compared to adult donor-derived platelets by profiling angiogenesis-related protein expression. Nineteen of 24 angiogenesis-related proteins were expressed equally, lower or higher in hiPSC-derived compared to adult platelets. The hiPSC-platelet’s coagulation hyporeactivity compared to adult platelets was confirmed by thromboelastometry. Further stepwise improvement of hiPSC-platelet production will, thus, permit better identification of platelet-mediated regenerative mechanisms and facilitate manufacture of sufficient amounts of functional platelets for clinical application.

## 1. Introduction

Platelets fulfill essential functions in hemostasis, thrombosis, innate immunity, vascular integrity, and regeneration after injury [1,2]. More than 100 million blood donations worldwide are necessary annually to meet the clinical need for blood products [3]. Approximately 1.5 million platelet transfusions are required to prevent bleeding or correct thrombocytopenia of <150 × 10^9^/L due to numerous conditions every year [4]. Various efforts have been made to overcome dependence on donor-derived platelets, in part due to the increasing need for blood products and decreasing blood donor availability according to demographic changes [5,6].

The demand of platelets in an adult, under steady state, was estimated at around 35 billion platelets/day [7,8]. Platelets share a common megakaryocyte erythroid progenitor (MEP) with red blood cells, which can produce mature megakaryocyte (MK) progeny. Within human bone marrow, at least 1000 platelets were estimated to be released per single MK over time. Several billion MKs are required to produce 2–3 × 10^11^ platelets for one unit of platelets for clinical use based on an estimated release of <50 platelets per MK in vitro [6]. Various methods have been tested to improve platelet production for research and therapy. CD34^+^ hematopoietic stem/progenitor cells (HSPCs) have been the traditional source for in vitro platelet propagation [9]. More recently, pluripotent embryonic stem cells (ESCs) and induced pluripotent stem cells (iPSCs) were recognized as a self-renewing source of all mature cell lineages including blood cells and particularly platelets [10,11]. Established 2D standard protocols for initiating platelet production use feeder-free culture of human iPSCs (hiPSCs) with either direct hemogenic endothelial cell (HEC) induction by bone morphogenetic protein 4 (BMP4) and basic fibroblast growth factor (bFGF) or vascular endothelial growth factor (VEGF) [12,13,14], or additional transcription factor gene transfer [15]. Accelerated Wnt-signaling via addition of the glycogen synthase kinase 3β inhibitor CHIR99021 (CHIR) during early mesoderm specification was shown to drive hiPSCs towards definitive hematopoiesis [16,17,18]. Alternative embryoid body (EB)-based protocols were established including sophisticated MK lineage forward reprogramming strategies [19,20,21]. A mesoderm bias might be expected in EBs, even in the absence of a dedicated mesoderm induction [22]. Human iPSC sacs were described to permit particularly efficient forward-programmed platelet propagation with a VEGF-only HEC induction protocol [23]. Several recent protocols took advantage of improved 3D bioreactor strategies [13,15,19,21,23]. A comprehensive overview of 3D MK and platelet culture protocols was published elsewhere [24,25]. In the majority of the protocols, the specification of hiPSCs towards MKs was obtained via exposure to the morphogen BMP-4 and the growth factor bFGF, with or without VEGF to initiate HEC formation [12,13,14,19,20,21]. Mesoderm induction support by CHIR-mediated Wnt signaling may also result in increased MK production [15].

We focused on platelet production due to our interest in the regenerative potential of human platelets [26]. We observed recently that mesoderm induction in advance of subsequent lineage specification was beneficial for several middle germ layer-derived cell types including stromal cells [27] and vascular endothelial cells [28]. We included activin A [29], BMP-4, CHIR, and VEGF as an initial two-day mesoderm induction step in our protocol. We found that this protocol significantly increased HEC differentiation resulting in improved MK production. Results were further validated by performing a direct parallel comparison of standard vs. improved platelet production in a selected efficient and affordable MK maturation medium. The coagulatory function of hiPSC-derived platelets was compared to adult donor-derived platelets by rotational thrombelastometry (ROTEM). The regenerative hiPSC-platelet proteome was multiplexed revealing a rich lineage-specific angiogenesis-related cargo.

## 2. Results

### 2.1. Improving HEC Differentiation and MK Progenitor (MKP) Production from hiPSCs

**Figure 1:**
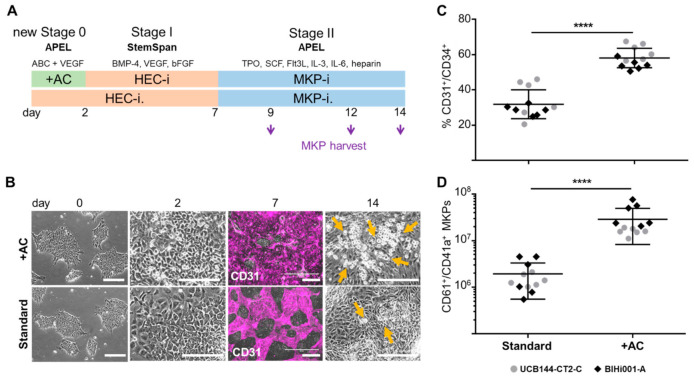
Production of hemogenic endothelial cells (HECs) and megakaryocyte progenitors (MKPs) from hiPSCs. (**A**) Experiment outline of hiPSC differentiation towards MKPs via HECs without (Standard) or with initial two-day (stage 0) induction including activin A and CHIR99021 (+AC) in APEL (Albumin Polyvinylalcohol Essential Lipids) medium containing activin A, bone morphogenetic protein-4 (BMP-4), CHIR99021 (ABC), and vascular endothelial growth factor (VEGF). Regular stage I and stage II media for HEC induction (HEC-i.) and MKP induction (MKP-i.) are composed as indicated (see Table 1). Floating MKPs were harvested on days 9, 12, and 14 as indicated by purple arrows. (**B**) Cell morphology documented on days 0, 2, 7, and 14 during hiPSC to MKP differentiation. Representative pictures from four replicates of clone UCB144-CT2-C are shown. In situ CD31 reporter staining on day 7 highlighting HECs (purple). Depicted reporter staining results corresponding to 66% and 20% CD31^+^/CD34^+^ cells with improved vs. standard conditions, respectively, as analyzed by flow cytometry (Appendix A Figure A1). On day 14, round-shaped floating cells (indicated by orange arrows) were more prominent after initial induction (+AC). Scale bars: 200 µm. (**C**) CD31^+^/CD34^+^ cells as detected by flow cytometry after improved compared to standard conditions on day 7 (*n* = 12; *p* < 0.0001). (**D**) CD61^+^/CD41a^+^ MKPs after induction (+AC) per 1 × 10^6^ starting hiPSCs compared to standard conditions (*n* = 12; *p* < 0.0001). Results from hiPSC clones UCB144-CT2-C (grey circles) and BIHi001-A (black diamonds); unpaired t-tests, **** *p* < 0.0001 (**C**,**D**).

**Figure A1:**
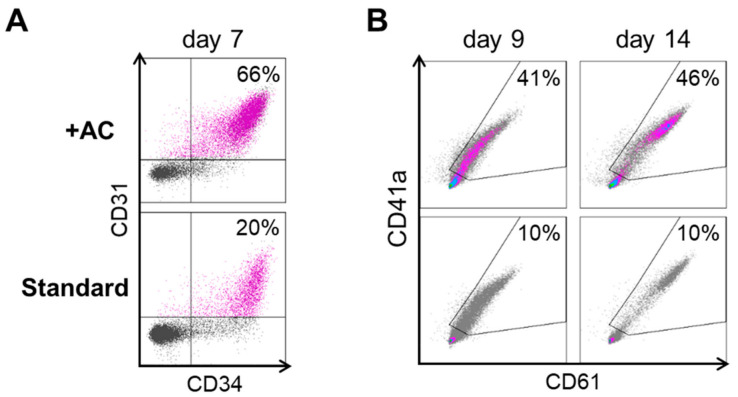
Representative flow cytometry plots of (**A**) hiPSC-derived HECs corresponding to Figure 1B day 7 reporter staining and (**B**) MKPs.

In a first series of experiments, we compared HEC induction from two independent randomly selected hiPSC strains with and without an initial two-day induction phase (Figure 1A)**.** Cells differentiation under improved conditions assumed a more dense appearance at day 2 before replacing the induction medium by standard HEC induction medium. On day 7, in situ reporter staining using an anti-human CD31 antibody [28] revealed more HECs in cultures with initial induction (Figure 1B). A significant increase of CD31^+^/CD34^+^ cells was detected by flow cytometry compared to standard conditions on day 7 (at the end of stage I; Figure 1C and Appendix A Figure A1). On day 14, an increased amount of round-shaped floating cells was observed in cultures after initial induction (Figure 1B). During stage II, floating cells derived from the culture supernatants were harvested, counted, and analyzed by flow cytometry. The total count of CD61^+^/CD41a^+^ MKPs per 1 x 10^6^ input hiPSCs was significantly increased to 29 ± 21 × 10^6^ compared to standard conditions with 1.9 ± 1.4 × 10^6^ (both mean ± SD; Figure 1D).

### 2.2. Expansion, Maturation and Platelet Production of hiPSC-MKs in Different Media Conditions

**Figure 2:**
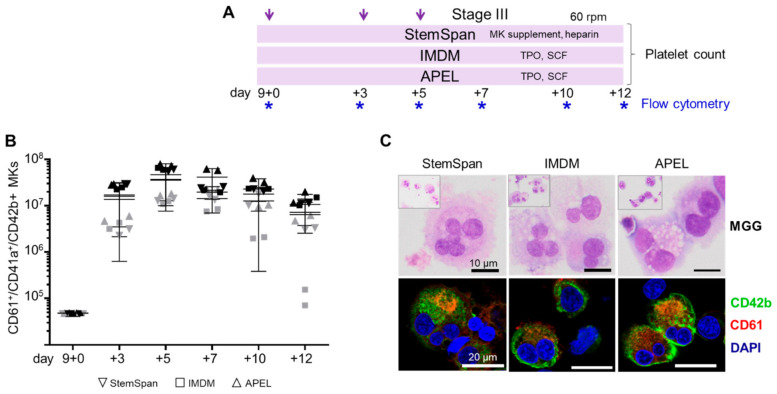
Expansion and maturation of hiPSC-derived megakaryocytes (MKs) in different media conditions. (**A**) Experimental outline using the improved induction conditions. Megakaryocyte progenitors (MKPs) derived from stage II at days 9, 12, and 14 (purple arrows) were harvested and matured into MKs in stage III for up to 12 additional days in three different media (StemSpan, IMDM or APEL), supplemented as indicated. From day 9 + 10 on, MKs were cultured under dynamic conditions at 60 rpm. Cells were harvested, counted, and analyzed by flow cytometry (platelet counts are shown in Figure 3B,C). (**B**) The total amount of CD61^+^/CD41a^+^/CD42b^+^ maturing MKs revealed no significant difference after culture in the three different media (StemSpan, down pointing triangle; IMDM, squares; APEL, upward pointing triangles; three different mean value lines representing the three media; symbol color depicts the two independent hiPSC clones UCB144-CT2-C, grey and BIHi001-A, black; *n* = 4, *p* > 0.05; two-way ANOVA, multiple comparisons). (**C**) MKs showed various maturation stages, indicated by two or more nuclei (deep purple), independent of media conditions (May–Grünwald Giemsa stain, MGG; top row, lower magnification inserts). Characteristic MK immunophenotype CD42b (green), CD61 (red); nuclear DAPI stain (blue; lower row). Representative pictures of two replicates of clone BIHi001-A are shown after harvest on day 9 + 7 (stage III) and stained as specified.

We next asked whether MKPs generated by the improved protocol respond to standard maturation conditions by producing mature MKs. We selected two frequently used reference media supplemented with thrombopoietin (TPO) plus stem cell factor (SCF) and a commercial kit containing an ‘MK supplement’ plus heparin [12,30,31]. The final stage III culture phase beyond day 9 + 9 was done on an orbital shaker at moderate rotation (60 rpm) to induce shear stress supporting platelet release (Figure 2A).

**Figure 3:**
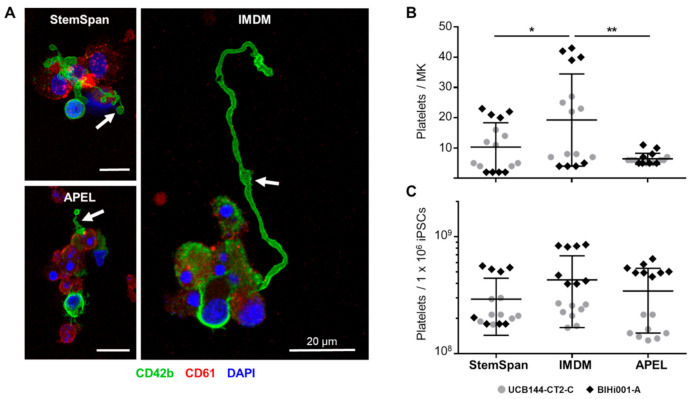
Human iPSC-derived megakaryocytes (MKs) produce pro-platelets and release platelets in all media conditions. (**A**) MKs with pro-platelet structures on day 9 + 12 (stage III) after dynamic culture in supplemented StemSpan, IMDM, and APEL media. Representative immunofluorescence images of MKs from one hiPSC clone (UCB144-CT2-C) stained with CD42b (green), CD61 (red), and DAPI (nuclei, blue) are shown. Pro-platelets are indicated by white arrows. (**B**) Platelet counts on day 9 + 12 (stage III) calculated per input MK were significantly increased after culture in supplemented IMDM medium, compared to StemSpan (*p* = 0.046) and APEL medium (*p* = 0.0273; both unpaired t-tests * *p* < 0.05 and ** *p* < 0.01). (**C**) Additional analysis showed comparable platelet counts per 1 × 10^6^ input hiPSCs (*p* > 0.05) in all media conditions. Results from two independent hiPSC clones (UCB144-CT2-C, grey circles, and BIHi001-A, black diamonds; *n* = 8, measured in duplicates).

The hiPSC-derived HECs differentiated into CD61^+^/CD41a^+^/CD42b^+^ MKs irrespective of basic media conditions. A trend towards lower differentiation efficiency of the cord blood-derived hiPSC clone was observed over time. Up to 33 ± 12 × 10^6^ MKs (mean ± SD) per 1 × 10^6^ input hiPSCs were obtained with peak levels around days 5–7 of the stage III culture (Figure 2B). The multinucleated MKs expressed glycoprotein Ib (CD42b) and integrin β3 (CD61) under all three media conditions tested (Figure 2C). Pro-platelet formation was observed in all three media conditions at the end of stage III culture (cumulative day 9 + 12; Figure 3A). We obtained 4–43 platelets at the end of culture stage III in TPO/SCF-supplemented IMDM (19 ± 15) compared to 5–11 platelets in APEL-based (6 ± 2) and 2–23 platelets in StemSpan-based (10 ± 8; all mean ± SD) media conditions (Figure 3B). We also observed certain experimental variability and no significant difference in the number of platelets generated per 1 x 10^6^ input hiPSCs (290 ± 150 × 10^6^, 430 ± 260 × 10^6^ and 340 ± 190 × 10^6^ in StemSpan, IMDM and APEL-based media, respectively; mean ± SD; Figure 3C). 

### 2.3. Validation That the Modified Protocol Improves Platelet Production from hiPSCs

**Figure 4:**
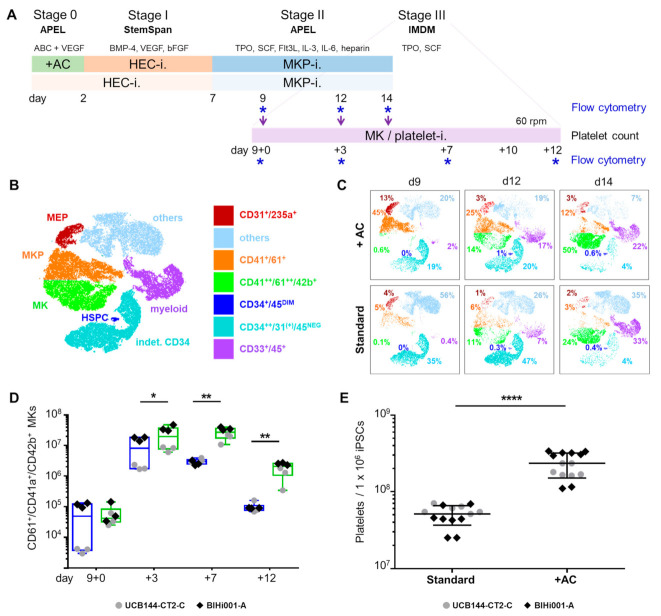
Validation of improved platelet production from hiPSCs. (**A**) Platelet production from hiPSCs comparing the improved protocol (stage 0, +AC) followed by hemogenic endothelial cell induction (HEC-i., stage I) and megakaryocyte progenitor induction (MKP-i., stage II) to a standard protocol, both finished with platelet production in IMDM supplemented with TPO and SCF (stage III). From day 9 + 10 on, MKs were cultured under dynamic conditions on an orbital shaker at 60 rpm. Cells were analyzed by flow cytometry. (**B**) Color code legend for t-distributed stochastic neighbor embedding (tSNE) plots defining MKs, MKPs, megakaryocyte erythroid precursors (MEP), myeloid cells (myeloid), indetermined CD34^+^/45^NEG^ cells (indet. CD34; reduced CD31 expression compared to early HECs) and other cells not allocated to main hematopoietic populations within the 10-color flow cytometry panel (others). Phenotypic identifiers as depicted in the legend insert. (**C**) Individual tSNE plots for stage II MKP culture constituents measured on days 9, 12, and 14 in cultures with (+ AC) and without initial induction, respectively. Results are depicted using the same tSNE color code. (**D**) The total amount of CD61^+^/CD41a^+^/CD42b^+^ MKs was significantly increased on day 9 + 3 (*p* = 0.0191), day 9 + 7 (*p* = 0.0037) and day 9 + 12 (*p* = 0.0052; paired t-tests) using the improved protocol (green boxes) compared to standard conditions (blue boxes). Results from two independent hiPSC clones (*n* = 6). (**E**) Platelet counts per input hiPSC on day 9 + 12 were significantly increased using the improved protocol (*p* < 0.0001, unpaired *t*-test). Symbols depict two independent hiPSC clones (UCB144-CT2-C, grey circles and BIHi001-A, black diamonds, *n* = 7; measured in duplicates in **D** and **E,** * *p* < 0.05, ** *p* < 0.01 and **** *p* < 0.0001).

Based on the results described so far, we selected the IMDM-based stage III medium conditions for a final validation experiment comparing cultures with and without initial activin A and CHIR-containing induction in parallel in one pass (Figure 4A). We used 10-color flow cytometry to create high content single cell-based data throughout MK differentiation. Datasets from stage II cultures with or without initial induction at culture days 9, 12, and 14, respectively, of a representative experiment, were accumulated for t-distributed stochastic neighbor embedding (tSNE) defining major single cell-based phenotype map (Figure 4B). 

Applying the tSNE map to individual culture analyses demonstrated the continuous development of MKs at the expense of MKPs over time. Cultures using the improved protocol showed 45% MKPs already at day 9 (stage II) compared to only 5% in standard cultures. Until day 14, the MK content rose to 50% in improved cultures, accompanied by a marked reduction in MKPs, and to 24% MKs with minimum decline in MKPs in standard cultures in this representative experiment, respectively. Results recapitulated the significantly higher amount of MKs in improved cultures compared to parallel standard cultures. We observed an almost disappearance of CD235a^+^ MEPs and HECs, and an increase of myeloid progenitors in both protocols to 22% and 33%, respectively, over time.

**Figure A2:**
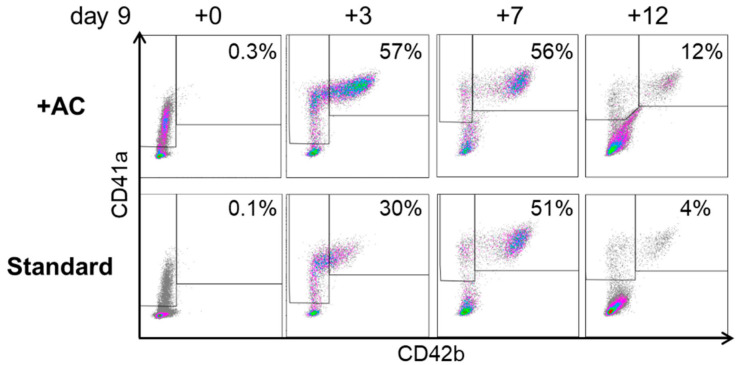
Representative flow cytometry dot plots of megacaryocyte maturation with (top row, +AC) or without initial induction (bottom row, Standard) of UCB144-CT2-C hiPSCs during stage III culture analysed for CD41a/CD42b reactivity at indicated time points.

**Figure A3:**
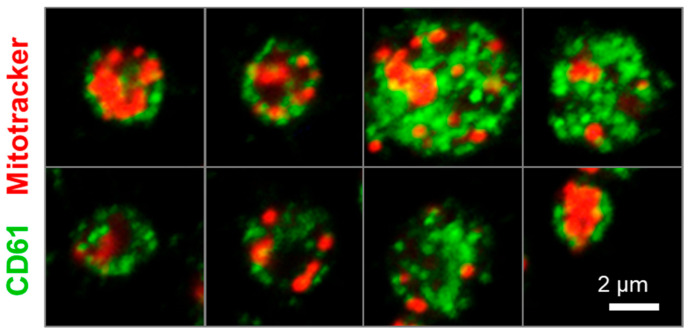
Illustration of hiPSC-derived platelet phenotype. The hiPSC-derived platelets were harvested d 9 + 7 from cultures using our improved protocol, fixed and stained for CD61 (green) and with mitotracker (red). Scale bar 2 µm. Representative platelets were depicted.

A small population of up to 1% CD34^+^/CD45^DIM^ HSPCs appeared under both conditions at later culture stages. Cultures in the improved protocol included the highest percentage of MKs and less unidentified ‘other’ cells compared to standard cultures (maximum 20% vs. maximum 56%, respectively) on day 14 (Figure 4C). Flow cytometry on day 9 + 7 confirmed that significantly more triple-positive CD61^+^/CD41a^+^/CD42b^+^ MKs were generated per 1 × 10^6^ starting hiPSCs using the improved protocol (27 ± 11 × 10^6^ MKs) compared to standard cultures (3.0 ± 0.6 × 10^6^ MKs; both mean ± SD) also in this validation experiment (Figure 4D). Representative flow cytometry dot plots of CD41a^+^/CD42b^+^ MKs during stage III culture are depicted in Appendix A Figure A2. As a consequence, significantly higher platelet counts, normalized per one million input hiPSCs, could be generated through improved initial induction (235 ± 84 × 10^6^ versus 51 ± 15 × 10^6^; mean ± SD) within 21 days (Figure 4E). The validation experiments confirmed the initial observation that equal amounts of 10–17 platelets were released per MK irrespective of the origin of MKs. These platelets showed typical round morphology with a 2–4 µm diameter, expressed CD61 and contained a variable amount of mitochondria (Figure A3).

### 2.4. Human iPSC-Derived Platelets Are Functional and Contain Angiogenesis-Related Proteins

**Figure 5:**
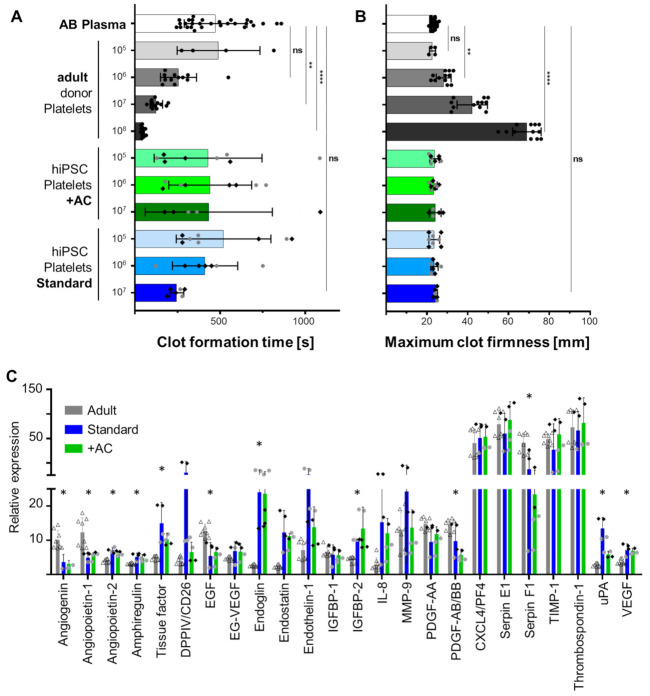
Analysis of coagulation and angiogenesis-related protein expression in adult healthy donor- and hiPSC-derived platelets. (**A**,**B**) Clot formation potential of standard culture vs. improved (+AC) hiPSC-derived platelets was compared to adult donor-derived platelets using rotational thromboelastometry (*n* = 4–12 platelet samples as indicated by individual result icons; *n* = 28 control plasma tests of the same AB plasma pool illustrating individual variability; hiPSC platelets derived from clones UCB144-CT2-C, grey circles and BIHi001-A, black diamonds; two-way ANOVA, multiple comparisons, ** *p* < 0.01 and **** *p* < 0.0001; ns, not significant). (**A**) Clot formation time induced by adding increasing numbers of platelets as indicated to 300 µL AB plasma control. (**B**) Maximum clot firmness in the absence (AB plasma) or presence of increasing number of platelets as indicated. Analysis was standardized to 2 × 10^7^ input platelets per membrane and measured in duplicate spots. (**C**) Several angiogenesis-related proteins were significantly increased (angiopoietin 2, *p* < 0.0001; amphiregulin, *p* = 0.0002; tissue factor, *p* = 0.0005; endoglin, *p* < 0.0005; endostatin, *p* < 0.0005; IGFBP2, *p* = 0.0004; VEGF, *p* < 0.0001) or decreased (angiogenin, *p* < 0.0001; angiopoietin 1, *p* = 0.0006; EGF, *p* < 0.0001; PDGF-AB/BB, *p* = 0.0014; serpin F1, *p* = 0.0001) in hiPSC-derived compared to adult platelets (grey bars, open triangles). No significant differences were detected between standard, (blue bars) and improved conditions (+AC, green bars). Results from two independent hiPSC lines (UC0144-CT2-C, grey circles and BIHi001-A, black diamonds) differentiated into platelets with both protocols were compared to four healthy donor-derived platelet preparations; analysis in duplicates. Multiple t-tests with Holm–Sidak correction (*n* = 4; * *p* < 0.05).

Testing of in vitro produced platelet function is challenging not only due to the risk of platelet activation during sample manipulation but also because of frequently limited hiPSC-derived platelet numbers [32]. Out of a series of contemporary methods we chose the viscoelastometric ROTEM point-of-care test to analyze the procoagulant potential of platelets. We have successfully implemented this method previously to estimate hemocompatibility of ex vivo propagated stromal cells [33]. Healthy adult donor-derived patelets reduced the clot formation time of pooled blood group AB plasma significantly in a dose-dependent manner. Both improved and standard culture hiPSC-derived platelets did not accelerate clot formation significantly due to highly variable results with only some preparations reducing the time to clot formation (Figure 5A). Maximum clot firmness was unaffected irrespective of the hiPSC-platelet dose added. Healthy adult donor platelets increased the maximum clot firmness dose-dependently with most significant effects at 10^8^ platelets per 300 µL test plasma, a level corresponding to a physiological platelet count of 333,000 platelets/µL (Figure 5B). 

We finally measured the content of angiogenesis-related proteins in platelets by antibody-based array analysis. Angiopoietin 2, amphiregulin, endoglin (CD105), endostatin, IGFBP2, and VEGF were significantly increased in hiPSC-derived platelets. Angiogenin, angiopoietin, epidermal growth factor (EGF), platelet-derived growth factor (PDGF)-AB/BB, and serpin F1 were significantly lower expressed in hiPSC-derived compared to adult donor platelets. The majority of angiogenesis-related proteins tested was equally expressed in adult and iPSC-derived platelets. Platelets derived from hiPSCs with both protocols showed comparable expression patterns. Tissue Factor was significantly higher expressed in both types of hiPSC-derived compared to adult donor-derived platelets (*p* = 0.0005; (Figure 5C). 

## 3. Discussion

This study demonstrates that distinct preceding induction by activin A, BMP-4, CHIR, and VEGF in lipid-enriched APEL medium resulted in a significant increase of hiPSC-derived platelet production using an otherwise unaltered three-stage standard protocol. As a result, we obtained mean 15 platelets per MK and mean 338 platelets per input hiPSC, respectively, over all experiments. This compares well to previously optimized published protocols. The result of the standard protocol obtaining mean 17 platelets per MK without previous induction (corresponding to mean 51 platelets/hiPSC) were comparable to two reference papers [12,13]. From a mechanistic point of view, the major effect observed after an initial induction step appeared to be a more robust HEC formation (stage I). We found nine-fold higher MKP proportions already at day 9 (stage II) of the improved protocol compared to standard cultures as illustrated in tSNE plots. This translated into a significantly higher number of MKs and platelets produced per hiPSC compared to standard conditions. We did not observe significant differences in the number of platelets produced by individual MKs irrespective of initial culture conditions. The rich angiogenesis-related proteome of hiPSC-derived platelets did not differ irrespective of initial induction. Significant differences between adult donor-derived and hiPSC-derived platelet proteomes may at least in part be donor-dependent requiring future extended analysis. 

We used 10-color flow cytometry throughout the study to identify and quantify differentiated hiPSC progeny. Monitoring differentiation in stage II in more detail using tSNE plots, based on the high amount of 12-parameter single cell data (10-color differentiation marker profiling and two scatters per single cell, at least 10,0000 cells analyzed per timepoint and condition), enabled comprehensive monitoring of the fate of multiple hematopoietic populations at once. These new data illustrated the progression of MKP maturation into MKs over time in stage II, accompanied by vanishing of MEPs. Appearance of HSPCs and myeloid progenitors at later time points of MKP cultures (stage II, days 9 and 12) might indicate lack of progenitor purity or limited efficiency of the TPO plus SCF-guided differentiation signaling rather than representing a reservoir for cells for continuous long-term platelet manufacture. This needs to be addressed by future investigation as this study had a clear focus on addressing the impact of mesoderm induction on hiPSC platelet production. The lower frequency of MKs under standard conditions reflects the reduced HEC input. 

Platelet manufacture for clinical purposes remains a challenge [12]. Assuming a maximum of 2000 platelets to be produced by one human bone marrow MK in vivo, 100 million MKs would be estimated to produce the equivalent of a single platelet concentrate comprising 2 × 10^11^ platelets for transfusion [9]. Starting from seven million hiPSCs at research scale in this study, we obtained only 1.64 ± 0.59 × 10^9^ platelets in total (mean ± SD). In a recent study, we demonstrated reproducible hiPSC 2D large scale propagation and obtained 9.8 ± 1.2 × 10^8^ hiPSCs (mean ± SD) from one million hiPSCs per four-layered cell factory on 2528 cm^2^ growth area within eight days [34]. MK culture using our improved protocol would suffice producing one single unit of platelets for transfusion including 2.5 × 10^11^ platelets, in theory, within less than a month. This will be tested in the near future before evaluating novel bioreactor-based, more complex and more expensive culture modalities. Earlier this year, an increased number of mature forward programmed MKs and platelets per MK were described after a 24-hour exposure to CHIR during mesoderm commitment [15]. However, the authors did not use CHIR in their final protocol. While preparing our manuscript, we realized that another group also included CHIR in their MK differentiation protocol from human embryonic stem cells (hESCs), producing a 0.6-fold higher MK yield to 7.3 ± 3.0 MKs/ hESC in an approach using suspension cell stack culture chambers compared to monolayer induction [35].

It is so far not clear which protocols and cell propagation devices are best suited for platelet mass-production [12]. We realized a certain level of variability in our 2D culture protocol, which leaves space for further improvement. Evidence is accumulating that 3D bioreactor-based culture will be considerably more efficient [19,23,36,37,38]. Mean 29.9 ± 10.9 MKs/hiPSC could be produced by combining hiPSC-aggregate cultivation in suspension culture with laminin 521-coated micro-carrier technology, compared to 3.9 ± 1.0 MKs/hiPSC obtained from cell-only aggregates (mean ± SD, respectively) in stirred spinner flasks bioreactors. After intravenous transfusion into immunodeficient mice, human platelets were released into the murine blood circulation by these MKs [19]. Comparable results were obtained in our study with the improved protocol. Sophisticated microfluidic organ-on-a-chip models are currently also under development enabling technically advanced bone marrow construction that may further advance platelet manufacture at clinical scale [13,39]. A nature-inspired system for functional MK and platelet production using silk-based vascular tubes was already introduced a decade ago [37]. With a more old-fashioned approach, roller-bottles were recently utilized to significantly enhance MK propagation from cord blood-derived CD34^+^ HSPCs by about 1.8-fold to 2.5 × 10^4^ MKs per initial CD34^+^ HSPC compared to static culture conditions; the number of platelets obtained was not disclosed in this study [40]. In our study, platelet release from MKs was supported by culturing on an orbital shaker during the final stage III. We plan to scale-up our protocol using bioprocessing in stirred bioreactors [41].

Functional assays are partly limited by the low numbers of platelets produced in vitro. Flow cytometry can be used for analysis of platelet surface and activation markers, but does not provide information about hemostatic potency of platelets [32]. We, therefore, plan to include platelet coagulation marker profiling in a follow-up study. In this study, we tested the pro-coagulant activity of hiPSC-derived platelets in plasma after activation with tissue factor by ROTEM, a viscoelastometric hemostasis assay commonly used as point of care test in the clinic [42]. We compared clot formation to donor derived platelets and observed the expected hyporeactivity of hiPSC-derived compared to healthy adult donor-derived control platelets. Unlike adult platelets, hiPSC-derived platelets were considered previously to be more similar to fetal or neonatal platelets, also showing hyporeactivity to stimulating agents [25,43]. This may be advantageous considering application of iPSC-derived platelets for intrauterine or neonatal transfusion in the future. The transfusion of hyporeactive iPSC-derived platelets avoiding adult donor-derived platelets may possibly overcome fetal or neonatal distress and some treatment-related complications [44,45]. At the moment, we can just speculate that higher tissue factor expression in hiPSC-derived platelets contributes mechanistically to their decreased susceptibility to activation by tissue factor in the ROTEM assay. This needs to be addressed in future studies. 

Another interest behind our efforts was the in vitro propagation of hiPSC-derived platelets as an ideally well-defined sustainable source of pro-angiogenic and regenerative factors for good manufacturing practice (GMP)-compliant cell production and regeneration [46,47]. Human platelet lysate (HPL) derived from donor platelets has replaced fetal bovine serum as a key cell culture supplement particularly for clinical-grade advanced therapy medicinal product (ATMP) manufacture [26,48]. 

We expect that hiPSC-platelet technology will contribute to understand the molecular mechanisms underlying the multiple functions of HPL [49]. Extracellular vesicles derived from platelets can mediate most of the regenerative effect of HPL during organoid formation and skin organ regeneration [28]. Platelet numbers in the range of >10^8^, which are currently obtained by many researchers in laboratory scale, will be sufficient to perform well-designed mechanistic studies towards better understanding regenerative platelet functions. Further improvements of hiPSC-derived platelet production technologies will consider GMP requirements [12,25,50] and might focus on using particularly histocompatible hiPSCs [12,14,25,51,52]. This will enable providing clinical platelet units, particularly for patients suffering from alloimmunization against frequent antigens, otherwise lacking suitable donors, in the near future.

## 4. Materials and Methods

### 4.1. Maintenance and Expansion of hiPSC Lines

Primary cell samples were collected from healthy volunteers after written informed consent according to the Declaration of Helsinki. Human iPSCs (UCB144-CT2-C) were generated by reprogramming primary umbilical cord blood stromal cells obtained with permission from the Institutional Review Board of the Medical University of Graz (protocols EK 19–252, EK 21–060), using a Cytotune™-iPS Sendai reprogramming kit (Thermo Fisher Scientific, Waltham, MA, USA), as described [27]. Dermal fibroblasts were reprogrammed to hiPSCs and characterized by the Stem Cell Core Facility, Charité, Berlin Institute of Health (BIHi001-A) (Human pluripotent stem cell registry (hPSC^reg®^). Available online: https://hpscreg.eu/cell-line/BIHi001-A, accessed on 10 June 2021). Human iPSCs were maintained under feeder-free conditions at 37 °C in 5% CO_2_, 5% O_2_ on Matrigel (Corning, NY, USA) in mTeSR1™ medium (Stemcell Technologies, Vancouver, CB, Canada) and routinely passaged as colonies using gentle cell dissociation reagent (Stemcell Technologies). 

### 4.2. Differentiation of hiPSCs into MKs and Platelets

For MK production, a 2D-based three-step standard differentiation protocol was used with modifications [12]. Feeder-free hiPSCs were harvested using Accutase^TM^ (Stemcell Technologies) and seeded at a density of 1 × 10^4^ cells per cm^2^ on Matrigel (Corning) in mTeSR1 containing 10 µM Y-27632 ROCK pathway inhibitor (Selleck Chemicals, Houston, TX, USA). After 24 h, hiPSCs were incubated for another day with mTeSR1 without Y-27632. Using the standard protocol, hiPSCs were cultured in stage I medium consisting of StemSpan^TM^-ACF (Stemcell Technologies) with BMP-4 (50 ng/mL, PeproTech, Rocky Hill, NJ, USA), bFGF (50 ng/mL, PeproTech) and VEGF (50 ng/mL R&D Systems, Minneapolis, MN, USA) for seven days. Alternatively, cells were incubated with StemDiff^TM^-Albumin Polyvinylalcohol Essential Lipids (APEL)2 (Stemcell Technologies)-based stage 0 medium [53], containing BMP-4 (30 ng/mL, PeproTech), activin A (25 ng/mL, PeproTech, Inc), VEGF (50 ng/mL, R&D Systems) and CHIR99021 (1.5 µM; Selleck Chemicals) as described [54], for two days, followed by an incubation with stage I medium for the five consecutive days. Medium was changed daily. On day 5, cells were passaged to 150 mm diameter cell culture plates (Greiner Bio-One, Kremsmünster, Austria). To obtain MKPs, HECs were incubated with stage II medium consisting of StemDiff^TM^ APEL2 (Stemcell Technologies) with TPO (25 ng/mL, Stemcell Technologies), SCF (25 ng/mL; PeproTech), FMS-like tyrosine kinase 3 ligand (Flt3L, 25 ng/mL; PeproTech), interleukin (IL)-3, IL-6 (10 ng/mL each; both Sigma-Aldrich, St. Louis, MO, USA), 5% protein-free hybridoma medium-II (PFHM-II, Thermo Fisher Scientific, Waltham, MA, USA), and heparin (5 IU/mL, Merck Millipore, Burlington MA, USA) for seven days. Supernatants containing MKPs were harvested on days 9, 12, and 14. In a final differentiation step, MKPs were cultured in MK/platelet maturation stage III medium containing Iscove’s Modified Dulbecco’s Medium (IMDM, Sigma-Aldrich) supplemented with 2-mercaptoethanol (50 µM, Thermo Fisher Scientific, Waltham, MA, USA), bovine insulin (10 µg/mL), human iron-free transferrin (5.5 µg/mL), sodium selenite (5 ng/mL), bovine serum albumin (0.5 mg/mL), linoleic acid (4.7 μg/mL, ITS+1, all Sigma-Aldrich), SCF (50 ng/mL, PeproTech) and TPO (20–50 ng/mL, Stemcell Technologies) [55] for up to twelve days at 0.5–1 × 10^6^ viable cells per mL. Where indicated, final maturation was performed with StemSpan^TM^ animal component free (ACF) medium (Stemcell Technologies) with 5 IU/mL heparin (Merck Millipore) and 1× StemSpan™ megakaryocyte expansion supplement (Stemcell Technologies) as described [12], or StemDiff^TM^ APEL2 (Stemcell Technologies) containing 5% PFHM-II (Thermo Fisher Scientific), SCF (50 ng/mL, PeproTech) and TPO (50 ng/mL, Stemcell Technologies) as described [14]. An overview on media conditions tested is given in Table 1.

### 4.3. Flow Cytometry

HECs, MKPs, and MKs were stained using a standard 10-color panel with FITC-conjugated anti-human CD61, PE-conjugated anti-human CD235a, APC-eFluor780-conjugated anti-human CD31, eFluor450-conjugated anti-human CD41a (10 µg/mL, clone HIP8, all eBioscience, San Diego, CA, USA), PerCP-Cy5.5-conjugated anti-human CD34 (2.5 µg/mL, clone 8G12), PE-Cy7-conjugated anti-human CD33 (1.25 µg/mL, clone P67.6), BV711-conjugated anti-human CD45 (2 µg/mL, clone HI30), BUV395-conjugated anti-human CD42b (4 µg/mL, clone HIP1, all BD Biosciences, Franklin Lakes, NJ, USA), and APC-conjugated anti-human CD309 (0.44 µg/mL, clone ES8-20E6, Miltenyi Biotec, Bergisch-Gladbach, Germany). Viability staining was performed in PBS at 4 °C for 15 min using FVS700 (3 ng/mL, BD Biosciences). Antibody staining was performed in brilliant stain buffer (BD Biosciences) for 20 min at 4 °C. Cells were analyzed on a five-laser (355, 405, 488, 561, and 637 nm) BD LSRFortessa™equipped with FACSDiva Software 8.0.1 firmware version 1.4 and Kaluza analysis software version 2.1.00002.20011 (Beckman Coulter, Brea, CA, USA).

T-distributed stochastic neighbor embedding (tSNE) analysis of a representative stage II differentiation time course experiment was done using FlowJo (v10.7.1) software (Beckmann Coulter). First, single viable cells from three time points (day 9, 12, and 14) of stage II with or without initial induction were gated and downsampled to 12,000 cells/sample and ‘concatenated’ to a single file afterwards. The tSNE analysis was performed using 2000 iterations, perplexity of 150 and learning rate (eta) of 5029. Calculated clusters were gated and populations identified by their marker expression.

### 4.4. Harvesting and Quantification of hiPSC-derived MKPs, MKs and Platelets

To obtain MKPs, supernatants derived from stage II were centrifuged at 100× *g* for 15 min. MKs and platelets were harvested by differential centrifugation using 100× *g* for 15 min to obtain MKs, followed by 1000× *g* for 15 min to precipitate platelets. The amount of trypan blue (Sigma-Aldrich) negative cells was determined in a Bürker-Türk counting chamber (BLAU BRAND^®^, Wertheim, Germany). The total count of CD61^+^/CD41a^+^ MKPs and CD61^+^/CD41a^+^/CD42b^+^ MKs was calculated from the percentages obtained after flow cytometry analysis. Platelets were quantified using an automated hematology analyzer (Sysmex KX-21N™, Sysmex Austria GmbH, Vienna, Austria).

### 4.5. Immunocytochemistry and May-Grünwald-Giemsa Staining

In-situ reporter staining after HEC differentiation was performed by incubating native cells with an AlexaFluor647-conjugated anti-human CD31 antibody (4 µg/mL, clone M89D, BD Biosciences) in basal medium for 30 min at 37 °C. MKs and platelets were fixed with 4% formaldehyde for 20 min at room temperature and cytospun at 550× *g* for 4 min onto coated Shandon™ cytoslides™ using a Shandon™ Cytospin 4 cytocentrifuge (both Fisher Scientific, Hampton, NH, USA). For immunocytochemistry, cells were washed in phosphate-buffered saline (PBS), permeabilized in citrate buffer, blocked with 1x Dako wash buffer (Agilent Technologies, Santa Clara, CA, USA) and supplemented with 10% fetal bovine serum (FBS, Lonza, Basel, Switzerland). Monoclonal anti-human CD42b (2 µg/mL, clone MM2/174, Novus Biologicals, Littleton, CO, USA) and anti-human CD61 (14 µg/mL, clone SJ19-09, Invitrogen, Waltham, MA, USA) primary antibodies were applied overnight at 4 °C. As secondary antibodies, goat anti-mouse IgG Alexa-Fluor 488 and goat anti-rabbit IgG AlexaFluor 555 (both Invitrogen) were applied for 1 hour at room temperature. Cell nuclei were stained with 4′,6-diamidin-2-phenylindol (DAPI, 0.2 µg/mL, Molecular Probes, Eugene, OR, USA). Mitochondria were stained with MitoTracker™ Red CMXRos (100 nM, Thermo Fisher Scientific) for 20 min in medium prior to fixation. May–Grünwald–Giemsa staining was performed by incubating the slides for 3 min in May–Grünwald solution (Carl Roth, Karlsruhe, Germany), followed by 10 min in Giemsa solution (1:20 diluted, Carl Roth). Slides were rinsed with distilled water, air-dried and mounted in quick-hardening mounting medium (Eukitt^®^, Sigma-Aldrich).

### 4.6. Image Acquisition

In situ reporter fluorescence-stained slides were analyzed on an EVOS^®^ FL microscope (Thermo Fisher Scientific). Confocal microscopy was performed using the laser scanning microscope Axio Observer Z1 attached to LSM700 (Carl Zeiss, Jena, Deutschland). Light microscopic cell culture pictures were obtained with an EVOS^®^ XL microscope (Thermo Fisher Scientific). Total slides were scanned automatically in 40 x magnification using the VS-120-L Olympus slide scanner 100-W system and processed using the Olympus VS-ASW-L100 program (Olympus, Shinjuku, Tokio, Japan).

### 4.7. Proteome Profiler Array

The presence of angiogenesis-related proteins in hiPSC-derived and healthy blood donor-derived platelets was detected using a proteome profiler array (proteome profiler human angiogenesis array kit, R&D Systems) according to the manufacturer’s instructions with loading of 2 × 10^7^ platelets per membrane. In brief, platelets derived from hiPSC-differentiation or from platelet concentrates, routinely produced at our blood center from healthy blood donors, were centrifuged at 1000× *g* for 15 min with prostaglandin E1 (PgE1, 1 µM, Sigma-Aldrich). Platelets were lysed with RIPA buffer (Sigma-Aldrich), supplemented with Halt™ Protease and phosphatase inhibitor cocktail (Thermo Fisher Scientific) and sonicated for 60 seconds on ice. For proteome profiler analysis, membrane spots were visualized and quantified using a Chemidoc system and imaging lab software 6.0.1 (all Bio-Rad Laboratories, Hercules CA, USA). Background signals were subtracted and signals were normalized compared to the reference spots located on the arrays.

### 4.8. Rotational Thromboelastometry

ROTEM^®^ (Tem^®^ International GmbH, Germany) was used as in vitro coagulation assay. Briefly, 20 µL calcium chloride solution reagent (Tem^®^) and 20 µL ex-tem^®^ reagent containing tissue factor (Tem^®^) were pipetted into a pre-warmed 37 °C cup using an automated pipette as indicated by ‘on screen’ instructions. Various amounts of adult platelets derived from healthy blood donors after written informed consent and iPSC derived platelets (1 × 10^5^, 1 × 10^6^, 1 × 10^7^, 1 × 10^8^, respectively) diluted in 300 µL citrated blood group AB plasma (pool of 10 donations) were added and analyzed via EXTEM^®^ assay for 30 min. Clot formation time (until 20 min) and maximum clot firmness were evaluated. Pure citrated blood group AB plasma was used instead of citrated whole blood as a reference as described [33].

### 4.9. Statistical Analysis

Statistical analysis was performed in GraphPad Prism version 6.05 (GraphPad Software, San Diego, CA, USA), using unpaired or paired student’s *t*-tests or two-way ANOVA analysis with multiple comparison, as indicated. Differences were considered statistically significant with a *p*-value < 0.05. 

## 5. Conclusions

Platelet production from hiPSCs or other stem/progenitor cell types and cell lines offers great opportunities providing human platelets for regenerative medicine and particularly in selected medical indications for patients lacking suitable donors. This study demonstrates that an improved protocol can further ameliorate platelet production from hiPSCs. We introduced tSNE analysis of polychromatic flow cytometry results for better monitoring hiPSC-MK maturation. ROTEM coagulation analysis confirmed the immature functional state of hiPSC-derived platelets. The rich pro-angiogenic proteome of the hiPSC platelets indicated applicability in regenerative medicine in addition to the visionary use for transfusing patients in the near future. 

## Figures and Tables

**Table 1:** Media conditions.

Stage/Differentiation	Basal Medium	Supplements	Reference
0/incl. activin A + CHIR	StemDiff™ APEL2	BMP-4 (30 ng/mL)	[54]
		VEGF (50 ng/mL)	
		activin A (25 ng/mL)	
		CHIR99021 (1.5 µM)	
I/HECs	StemSpan™ ACF	BMP-4 (30 ng/mL)	[12]
		VEGF (50 ng/mL)	
		bFGF (50 ng/mL)	
II/MKPs	StemDiff™ APEL2	TPO (25 ng/mL)	[12]
		SCF (25 ng/mL)	
		Flt3L (25 ng/mL)	
		IL-3 (10 ng/mL)	
		IL-6 (10 ng/mL)	
		heparin (5 U/mL) PFHM-II (5%)	
III a/platelets	StemSpan™ ACF	StemSpan™ megakaryocyte expansion supplement (1x)	[12]
		heparin (5 U/mL)	
III b/platelets	IMDM	ITS+1	[55]
		2-mercaptoethanol (50 µM)	
		SCF (50 ng/mL)	
		TPO (20–50 ng/mL)	
III c/platelets	StemDiff™ APEL2	PFHM-II (5%)	[14]
		SCF (50 ng/mL)	
		TPO (50 ng/mL)	

**Abbreviations:** HECs, hemogenic endothelial cells; MKPs, megakaryocyte progenitors; BMP-4, bone morphogenetic protein 4; VEGF, vascular endothelial growth factor; bFGF, basic fibroblast growth factor; TPO, thrombopoietin; SCF, stem cell factor; Flt3L, FMS-like tyrosine kinase 3 ligand; IL, interleukin; PFHM, protein-free hybridoma medium; ITS+1, insulin, transferrin, sodium selenite, + linoleic acid. Three different platelet production media (III a–c) were compared in an initial series of experiments.

## Data Availability

n.a.
